# Latency period of aristolochic acid-induced upper urinary tract urothelial carcinoma

**DOI:** 10.3389/fpubh.2023.1072864

**Published:** 2023-03-09

**Authors:** Jing-Rong Jhuang, Po-Chun Chiu, Tung-Che Hsieh, Chung-Hsin Chen, Yeong-Shiau Pu, Wen-Chung Lee

**Affiliations:** ^1^Institute of Epidemiology and Preventive Medicine, College of Public Health, National Taiwan University, Taipei, Taiwan; ^2^Taiwan Cancer Registry, Taipei, Taiwan; ^3^Department of Public Health, College of Public Health, National Taiwan University, Taipei, Taiwan; ^4^Department of Urology, National Taiwan University Hospital, Taipei, Taiwan

**Keywords:** aristolochic acid, upper urinary tract urothelial carcinoma (UTUC), latency period, time-varying effect, slope-change model

## Abstract

**Purpose:**

Aristolochic acid (AA) is a carcinogen in upper urinary tract urothelial carcinoma (UTUC). This study investigated the latency period between AA exposure and UTUC development.

**Materials and methods:**

This population-based cohort study was designed using record linkage of the National Health Insurance Research Database (NHIRD), Taiwan Cancer Registry Dataset, and cause-of-death data in Taiwan. Those aged 40–79 years were enrolled in this study. Patients who died or had renal insufficiency or UTUC before 2005 were excluded. The doses of AA exposure and rates of comorbidities between 2000 and 2005 were obtained. The Cox proportion hazard model was used to estimate the risk of UTUC between 2005 and 2016. In addition, the Cox model with time-varying coefficient of AA was used to measure the latency period of UTUC.

**Results:**

Of the 752,232 participants enrolled from the NHIRD, 520,871 (68.29%), 210,447 (27.59%), and 31,415 (4.12%) were exposed to cumulative AA doses of 0–1 mg, 1–150 mg, and >150 mg, respectively. A total of 1,147 (0.15%) patients were diagnosed with UTUC between 2005 and 2016. The latency periods of UTUC in middle-aged (40–59 years old) men with cumulative AA doses of 1–150 mg and middle-aged women with cumulative AA doses of 1–150 mg and >150 mg were 8, 9, and 7 years, respectively. Among the aged (60–79 years) individuals, no time-varying effect was observed, and the latency period could not be measured.

**Conclusion:**

A decreased risk of UTUC was observed after the ban on AA in Taiwan, especially in middle-aged women with moderate to high doses of AA exposure and men with moderate doses of AA exposure. The latency period of UTUC varies with age, the dose of AA exposure, and sex.

## 1. Introduction

Aristolochic acid (AA) was identified as a nephrotoxin based on the unusual finding that a cluster of young Belgian women presented with rapidly progressive renal failure after consuming a slimming herbal remedy containing AA (1, 2). Furthermore, Nortier et al. (3) reported that 95% of these women were observed to have either urothelial carcinoma (UC) or urothelial dysplasia in their native kidneys. Therefore, AA was not a nephrotoxin but also a carcinogen in this Belgium cohort. In some villages in Balkan, the population has consumed bread made from flour contaminated with seeds of *Aristolochia clematitis* for decades (4). Based on clinical and molecular evidence, AA was also demonstrated to be responsible for endemic nephropathy and upper urinary tract urothelial carcinoma (UTUC) in Balkan patients. In addition, nationwide exposure to AA from Chinese herbal remedies was observed in Taiwan. Between 1997 and 2003, at least 39% of Taiwanese people ingested AA-containing herbal products (AA-CHP) (5). AA-induced UTUC was confirmed in a considerable portion of UTUC patients according to the identification of both aristolactam-DNA adducts in the renal cortex and the signature mutation pattern in UTUC tumors in Taiwan (6). Using the nationwide health insurance database, those who took more AA-CHP were at higher risk of both UC (7) and end-stage renal disease (8).

Since the association between AA, renal failure, and UTUC has been confirmed, several countries have banned the prescription of AA-CHP, including Guangfangji (*Radix Aristolochiae Fangchi*), Guanmutong (*Caulis aristolochiae Manshuriensis*), Madouling (*Fructus Aristolochia*), Qingmuxiang (*Radix Aristolochiae*), Tianxianteng (*Herba Aristolochia*), and Zhushalian (*Radix Aristolochiae Tuberosa*) (9–11). This action limited the exposure of AA from major sources and might reduce the development of either renal insufficiency or UTUC in the population. Although the health authority in Taiwan banned the use of the abovementioned major types of AA-CHP in 2003, the incidence of UTUC continued to increase until 2010 (12). It reflects the carcinogenic effects of AA may persist long after the discontinuation of AA usage. However, there are no studies that represent the latency period of AA in the development of UTUC.

Wang et al. identified that the prescription frequency of AA-CHP in Taiwan for patients with end-stage renal disease decreased to nearly zero after 2005 (13). The combination of nationwide exposure to AA and the clear stop date of AA use makes Taiwan an ideal model to investigate the latency period. Therefore, this study was designed to explore the latency period in populations exposed to varied doses of AA using a nationwide registry database.

## 2. Materials and methods

**Figure 1:**
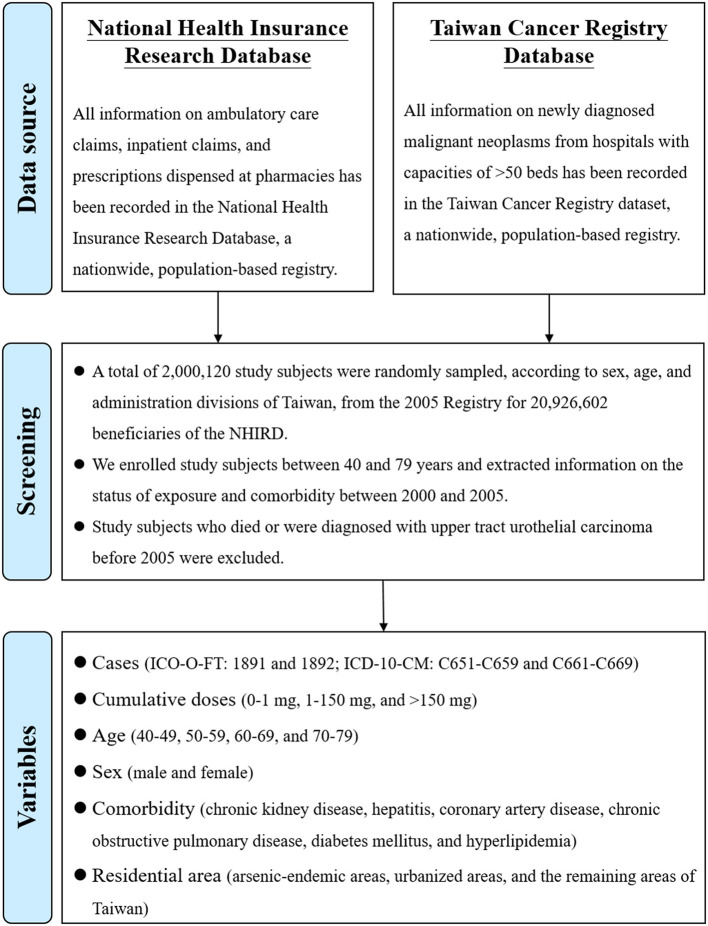
Flowchart for recruitment of study subjects.

This study was reviewed and approved by the Research Ethics Committee at National Taiwan University Hospital (201912201W). A population-based retrospective cohort study was designed using record linkage of the National Health Insurance Research Database (NHIRD) (14), the Taiwan Cancer Registry Dataset (TCRD) (15, 16), and cause-of-death data. The datasets used were retrieved from the Health and Welfare Data Science Center of the Ministry of Health and Welfare in Taiwan. A flowchart for the recruitment of study participants is shown in Figure 1. The study period was from January 2000 to December 2016. We conducted stratified random sampling according to sex, age, and administration divisions. Sex was divided into men and women. Age was divided into 20 groups at 5-year intervals from age 5 to 85. The region is classified into 6 categories based on the separate Divisions of the National Health Insurance Administration. A total of 240 subgroups (2 × 20 × 6) were created, and then the number of samples was drawn from each subgroup based on random sampling. A total of 2,000,120 participants were sampled from the 2005 registry for 20,926,602 beneficiaries of the NHIRD in Taiwan. In addition, we collected data including the status of AA exposure and comorbidities between 2000 and 2005, and enrolled study subjects aged between 40 and 79 years in 2005 (when the follow-up started). Subjects aged 80 years or older were not enrolled because of the conflict of competing risks of death. Participants who died or had been diagnosed with UTUC before 2005 were excluded from the study because of the unclear time-sequence between AA and UTUC.

Since 2003, the Ministry of Health and Welfare in Taiwan has banned the following herbs containing AA: (7, 12) Madouling (*Fructus Aristolochiae*), Tianxianteng (*Caulis Aristolochiae*), Guanmutong (*Aristolochia Manshuriensis*), Guangfangji (*Aristolochia Fangchi*), and Qingmuxiang (*Radix Aristolochiae*). A total of 923 Chinese herbal remedies containing these herbs were evaluated in this study. The study participants who used these AA-CHPs were identified from the NHIRD. The original amount of herbs and the total dose of AA for each AA-CHPs were determined using the detailed composition of AA-CHPs obtained from the Department of Chinese Medicine and Pharmacy, Ministry of Health and Welfare in Taiwan. The estimated average doses of AA per 1 g of Guanmutong, Guangfangji, Madouling, Qingmuxiang, and Tianxianteng are 2.59, 2.04, 0.63, 0.009, and 0.026, respectively (17–19). We calculated the estimated cumulative dose of AA exposure (CDAE) from 2000 to 2005 for each subject and categorized them into a reference group (0–1 mg) and two exposure groups (1–150 mg and >150 mg) for the comparability among the published studies (7).

Potential confounding factors, including demographic characteristics, living in arsenic-endemic areas or urbanized areas, and comorbidities, were considered and adjusted in the study. Study subjects were categorized into four age groups (40–49, 50–59, 60–69, and 70–79 in 2005 when the follow-up started). Arsenics was confirmed to be a carcinogen for urothelial carcinoma (20) and presented as a confounding factor in the identification of UTUC in Taiwan. A total of 21 townships in Taiwan (Supplementary Figure 1) where the concentration of arsenics in well water exceeded 0.35 ppm were defined as arsenic-endemic areas (21, 22). A total of 27 townships in Taiwan with a population size exceeding 20,000 and population density exceeding 300 per km^2^ were defined as urbanized areas (Supplementary Figure 1). Comorbidities were collected from the NHIRD and defined by the following diagnoses: renal insufficiency (ICD-9-CM code: 585), hepatitis (ICD-9-CM code: 070), coronary artery disease (ICD-9-CM code: 414), chronic obstructive pulmonary disease (ICD-9-CM codes: 490–496), diabetes mellitus (ICD-9-CM codes: 249–250), and hyperlipidemia (ICD-9-CM code: 272). The UTUC cases (ICO-O-FT: 189.1 and 189.2; ICD-10-CM: C65.1–C65.9 and C66.1–C66.9) from 2005 to 2016 were collected from the TCRD.

Characteristics of study participants were presented as categorical variables and compared using the chi-square test and Cochran–Armitage test for trend. Multivariable analyses of UTUC risk between 2005 and 2016 were conducted using the Cox proportional hazards model. Because the hazard of UTUC might change after the discontinuation of AA, Cox models with a time-varying coefficient (23) of CDAE were constructed to measure the latency period. Specifically, we assumed a piecewise constant hazard function for the hazard ratios of CDAE, segmenting the calendar year into four consecutive periods (2005–2007, 2008–2010, 2011–2013, and 2014–2016) to depict the temporal change in adjusted hazard ratios (aHR). Besides, assuming that prohibiting the use of AA-CHPs would affect the trend in the UTUC occurrence after h years, we considered the slope-change model as follows:


$$\begin{array}{l} \lambda \left(t|X,Z;\beta_{0},\beta_{1},\gamma \right)=\lambda_{0}\left(t\right)\times \exp\left(f{\left(\beta_{0},\beta_{1},t\right)}^{T}X+\gamma^{T}Z\right)\;and\\ \;f\left(\beta_{0},\beta_{1},\;t\right)=\beta_{0}+\beta_{1}\times (t-h+1)\times I(t\geq h-\;1), \end{array}$$

where X is a vector of exposure groups, Z is a vector of confounding factors, and *I*(.) is an indicator function. The model parameters include λ_0_(*t*) is the baseline hazard function, β_0_ is the baseline effect of exposure, β_1_ is the slope-change effect of exposure after h years, and γ is the effect of confounding factors. We regarded the “h” as the latency period. Subsequently, we performed model selection using the lowest Akaike information criterion (AIC) to determine the latency period. The latency period was defined as the duration between the exposure to AA and the identification of UTUC. The candidates included 11 slope-change models (*h* = 1, 2, …, 11) and a proportional hazard model. Statistical interactions were examined using the likelihood ratio test. Statistical significance was set at *p*-value < 0.05. All analyses were performed using SAS version 9.4 and R version 3.5.2.

## 3. Results

### 3.1. Patient population

A total of 752,232 participants aged 40–79 years were enrolled in this study from the NHIRD. The demographics of the study participants stratified by the CDAE between 2000 and 2005 are shown in Table 1. In this cohort, 520,871 (68.29%), 210,447 (27.59%), and 31,415 (4.12%) were exposed to CDAE of 0–1 mg, 1–150 mg, and >150 mg, respectively. More women (36.92%) had a CDAE of >1 mg than men (26.52%, *p* < 0.001). With increasing age, the proportion of participants with CDAE >1 mg reduced gradually (40–49 years: 32.41%; 50–59 years: 32.33%; 60–69 years: 31.35%; 70–79 years: 28.27%; *p* for trend < 0.01). Fewer study subjects had CDAE of >1 mg in the urbanized areas (26.96%) than in the non-urbanized areas (32.20%, *p* < 0.001). Fewer study participants had a CDAE of >1 mg in arsenic-endemic areas (26.47%) than in non-arsenic-endemic areas (31.80%; *p* < 0.001). Subjects with comorbidities had a significantly higher CDAE than those without comorbidities (*p* < 0.001). Among people with comorbidities, 58.58 and 13.61% took CDAE of >1 and >150 mg, respectively, during the study period.

**Table 1:** Characteristics of study participants from the National Health Insurance Research Database registry between 2000 and 2005.

	**Estimated cumulative doses of aristolochic acid from 2000 to 2005**			
	**0–1 mg**	**1–150 mg**	>**150 mg**	
	***N*** = **520,871**	***N*** = **210,447**	***N*** = **31,415**	***P*** **-value**
**Sex, No. (%)**				<0.001
Men	280,663 (73.48)	88,363 (23.14)	12,916 (3.38)	
Women	240,208 (63.08)	122,084 (32.06)	18,499 (4.86)	
**Age, No. (%)**				<0.001
40–49 years	219,718 (67.59)	91,263 (28.07)	14,113 (4.34)	
50–59 years	146,099 (67.67)	60,845 (28.18)	8,957 (4.15)	
60–69 years	89,431 (68.66)	35,664 (27.38)	5,165 (3.97)	
70–79 years	65,623 (71.74)	22,675 (24.79)	3,180 (3.48)	
**Urbanized area, No. (%)**				<0.001
No	468,301 (67.80)	193,489 (28.01)	28,969 (4.19)	
Yes	52,570 (73.04)	16,958 (23.56)	2,446 (3.40)	
**Arsenic-endemic area, No. (%)**				<0.001
No	511,537 (68.20)	207,501 (27.67)	31,001 (4.13)	
Yes	9,334 (73.53)	2,946 (23.21)	414 (3.26)	
**Comorbidity, No. (%)**				<0.001
No	499,312 (70.26)	187,040 (26.32)	24,332 (3.42)	
Chronic kidney disease	8,613 (69.26)	3,184 (25.61)	638 (5.13)	
Hepatitis	480 (24.01)	1,004 (50.23)	515 (25.76)	
Coronary artery disease	181 (31.87)	287 (50.53)	100 (17.61)	
Chronic obstructive pulmonary disease	8,259 (31.81)	13,555 (52.21)	4,149 (15.98)	
Hyperlipidemia	1,580 (32.57)	2,397 (49.41)	874 (18.02)	
Diabetes mellitus	2,446 (39.24)	2,980 (47.81)	807 (12.95)	

### 3.2. Univariable and multivariable analysis of UTUC risk

Between 2005 and 2016, 1,147 UTUC patients (0.15%) were diagnosed in this study cohort. Multivariable analysis was conducted based on the Cox proportional hazards model and presented in Table 2. The aHRs for all exposure groups (CDAE: 1–150 mg: aHR = 1.50, 95% CI 1.32–1.70; >150 mg: aHR = 2.21, 95% CI 1.77–2.77) were significantly higher than that in the reference group (0–1 mg). In addition, an apparent dose-response relationship (*p* for trend <0.01) was observed between the dose of exposure and the risk of UTUC. There was no significant difference in the UTUC risk between men and women (women: aHR = 1.09, 95% CI 0.97–1.22) and between living in the urbanized areas and non-urbanized areas (urbanized areas: aHR = 1.06, 95% CI 0.87–1.28). Older participants had a higher risk of UTUC than younger participants (reference: 40–49 years; 50–59 years: aHR = 3.07, 95% CI 2.49–3.77; 60–69 years: aHR = 7.91, 95% CI 6.49–9.62; 70–79 years: aHR = 10.24, 95% CI 8.34–12.57). The participants living in arsenic-endemic areas were at a higher risk (aHR = 1.53, 95% CI 1.07–2.17) of UTUC compared to those in non-arsenic-endemic areas. The participants with chronic kidney disease were at higher risk (aHR = 10.18, 95% CI 8.63–12.00) of UTUC compared to those without chronic kidney disease. There was no difference in UTUC risk between the participants with (aHR = 1.09, 95% CI 0.86–1.37) and without other comorbidities.

**Table 2:** Adjusted hazard ratios for the occurrence of upper tract urothelial carcinoma between 2005 and 2016, assuming proportional hazard and time varying effect of cumulative doses of aristolochic acid.

**Model type**	**No. of subjects**	**UTUC cases**	**Proportional hazard model**		**Slope-change model**	
			**Adjusted HR**	**95% CI**	**Adjusted HR**	**95% CI**
**Cumulative doses of aristolochic acid**						
**Baseline**						
0–1 mg	520,871	660	1.00	–	1.00	–
1–150 mg	210,447	398	1.50	(1.32–1.70)	1.48	(1.26–1.73)
>150 mg	31,415	89	2.21	(1.77–2.77)	2.52	(1.92–3.31)
**Slope change in 2012**						
0–1 mg	–	–	–	–	1.00	–
1–150 mg	–	–	–	–	1.00	(0.94–1.09)
>150 mg	–	–	–	–	0.89	(0.77–0.99)
**Sex**						
Men	381,942	514	1.00	–	1.00	–
Women	380,791	633	1.09	(0.97–1.22)	1.09	(0.97–1.23)
**Age**						
40–49 years	325,094	132	1.00	–	1.00	–
50–59 years	215,901	276	3.07	(2.49–3.77)	3.06	(2.49–3.77)
60–69 years	130,260	424	7.91	(6.49–9.62)	7.83	(6.34–9.53)
70–79 years	91,478	315	10.24	(8.34–12.57)	9.96	(8.12–12.22)
**Urbanized area**						
No	690,759	1,033	1.00	–	1.00	–
Yes	71,974	114	1.06	(0.87–1.28)	1.06	(0.87–1.29)
**Arsenic-endemic area**						
No	750,039	1,115	1.00		1.00	
Yes	12,694	32	1.53	(1.07–2.17)	1.52	(1.07–2.17)
**Comorbidity**						
No	710,684	896	1.00	–	1.00	–
Chronic kidney disease	12,435	174	10.18	(8.63–12.00)	9.85	(8.36–11.61)
Other comorbidities	39,614	77	1.09	(0.86–1.37)	1.09	(0.86–1.39)

### 3.3. Latency period in the whole population

**Figure 2:**
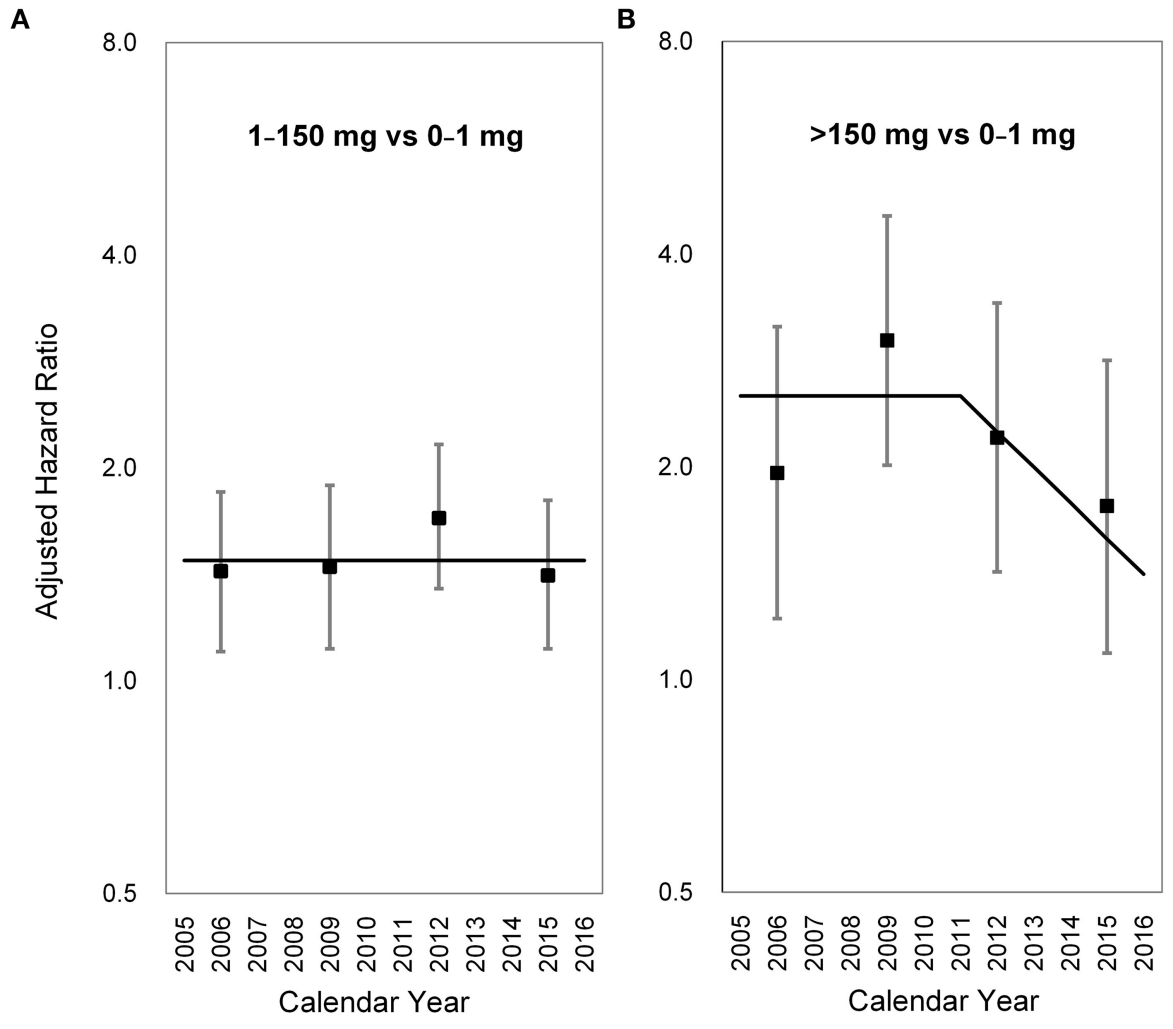
Long-term trends in the adjusted hazard ratio for upper tract urothelial carcinoma occurrence. **(A)** 1–150 mg vs. 0–1 mg, **(B)** >150 mg vs. 0–1 mg. Square points: adjusted hazard ratios from the piecewise constant hazard model. Vertical lines: 95% confidence intervals of the adjusted hazard ratios from the piecewise constant hazard mode. Solid lines: adjusted hazard ratios from the slope-change model.

To evaluate the latency period between AA exposure and the identification of UTUC, the slope-change model and the piecewise constant hazard model were constructed and presented in Tables 2, 3, respectively. Among subjects who took CDAE of 1–150 mg, no significant trend was observed in aHRs between 2005 and 2016 (Figure 2A). In contrast, among subjects with CDAE of >150 mg, the aHR increased from 1.96 (95% CI 1.22–3.16) in 2005–2007 to 3.02 (95% CI 2.01–4.53) in 2008–2010, and then gradually decreased to 1.76 (95% CI 1.09–2.83) in 2014–2016. The slope-change model showed that the aHR kept at 2.52 (95% CI 1.92–3.31) and decreased in 2011 (the 7th year after the ban on AA-CHPs; Figure 2B). The aHR will reach 1.00 in 2018–2019 based on the speed of 11% (95% CI 1–23%) per year.

**Table 3:** Adjusted hazard ratios for the occurrence of upper tract urothelial carcinoma between 2005 and 2016, based on the piecewise constant hazard model.

	**1–150 mg of aristolochic acid**		>**150 mg of aristolochic acid**	
	**Adjusted HR**	**95% CI**	**Adjusted HR**	**95% CI**
**The whole population**				
2005–2007	1.43	(1.10–1.85)	1.96	(1.22–3.16)
2008–2010	1.45	(1.11–1.89)	3.02	(2.01–4.53)
2011–2013	1.70	(1.35–2.16)	2.20	(1.42–3.41)
2014–2016	1.41	(1.11–1.80)	1.76	(1.09–2.83)
**Men aged 40–59 years**				
2005–2007	0.91	(0.41–2.01)	1.51	(0.36–6.39)
2008–2010	2.59	(1.55–4.33)	2.57	(0.90–7.31)
2011–2013	1.48	(0.81–2.69)	1.25	(0.30–5.25)
2014–2016	1.06	(0.58–1.95)	1.30	(0.48–2.49)
**Women aged 40–59 years**				
2005–2007	3.30	(1.31–8.34)	4.66	(1.61–13.46)
2008–2010	1.54	(0.63–3.79)	3.73	(1.65–8.41)
2011–2013	1.90	(1.00–3.60)	1.84	(0.71–4.78)
2014–2016	0.77	(0.36–1.68)	2.09	(0.92–4.73)
**Men aged 60–79 years**				
2005–2007	1.34	(0.82–2.21)	1.51	(0.55–4.19)
2008–2010	1.13	(0.66–1.93)	2.71	(1.23–5.99)
2011–2013	1.98	(1.26–3.12)	3.24	(1.53–6.88)
2014–2016	1.69	(0.99–2.91)	1.58	(0.49–5.12)
**Women aged 60–79 years**				
2005–2007	1.38	(0.94–2.02)	1.84	(0.88–3.84)
2008–2010	1.19	(0.73–1.94)	3.12	(1.52–6.39)
2011–2013	1.70	(1.16–2.48)	2.11	(1.00–4.42)
2014–2016	1.46	(0.99–2.15)	2.25	(1.11–4.53)

### 3.4. Latency period in the middle-aged population

**Figure 3:**
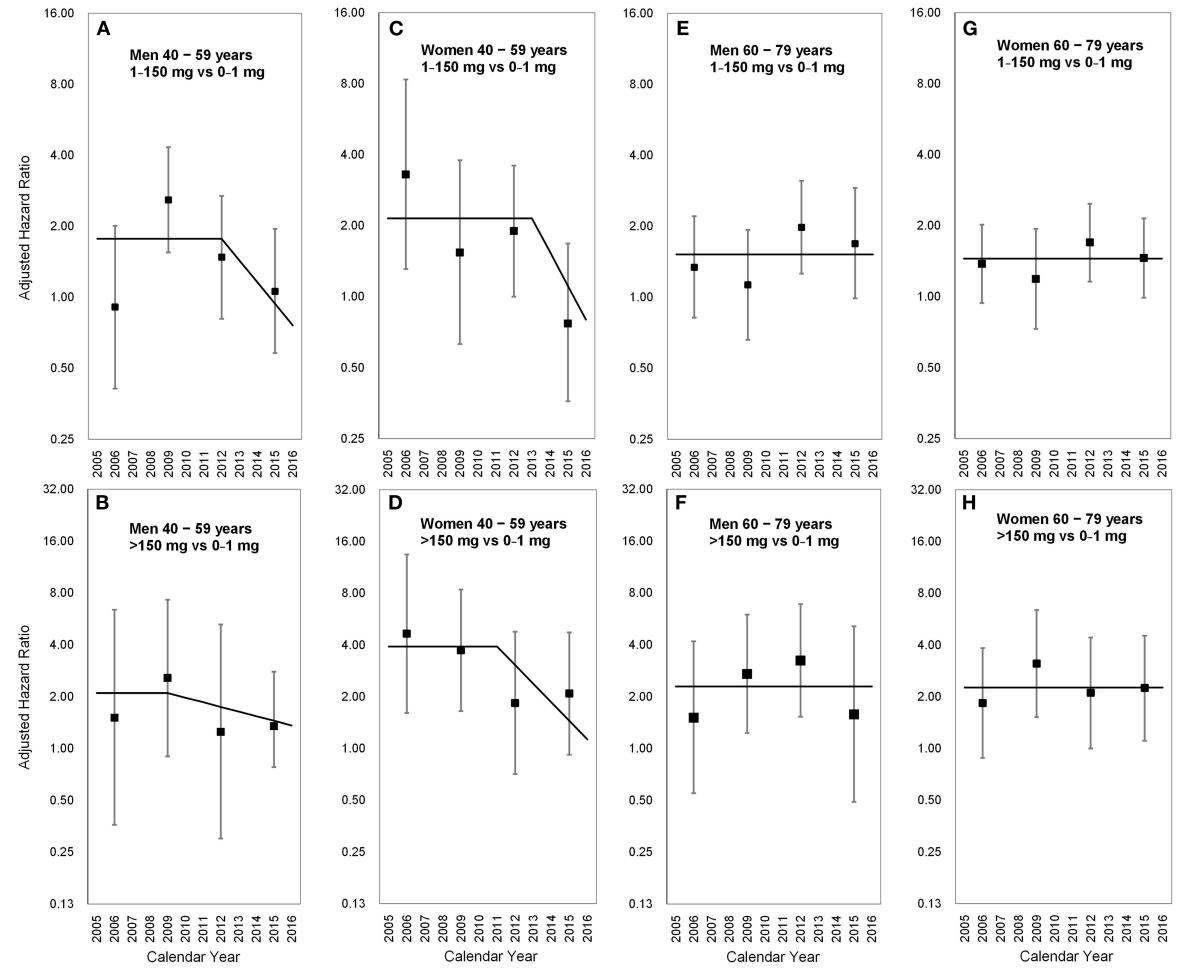
Long-term trends in the adjusted hazard ratio for upper tract urothelial carcinoma occurrence stratified by sex and age. **(A)** Men aged 40–59 years; 1–150 vs. 0–1 mg, **(B)** men aged 40–59 years; >150 vs. 0–1 mg, **(C)** women aged 40–59 years; 1–150 vs. 0–1 mg, **(D)** women aged 40–59 years; >150 vs. 0–1 mg, **(E)** men aged 60–79 years; 1–150 vs. 0–1 mg, **(F)** men aged 60–79 years; >150 vs. 0–1 mg, **(G)** women aged 60–79 years; 1–150 vs. 0–1 mg, **(H)** women aged 60–79 years; >150 vs. 0–1 mg. Square points: adjusted hazard ratios from the piecewise constant hazard model. Vertical lines: 95% confidence intervals of the adjusted hazard ratios from the piecewise constant hazard model. Solid lines: adjusted hazard ratios from the slope-change model.

The latency period varied with sex, age, and CDAE (*p* for interaction = 0.04). Among middle-aged men (40–59 years) who took CDAE of 1–150 mg, the aHR increased from 0.91 (95% CI 0.41–2.01) in 2005–2007 to 2.59 (95% CI 1.55–4.33) in 2008–2010, and then gradually decreased to 1.06 (95% CI 0.58–1.95) in 2014–2016. The slope-change model showed that the aHR kept at 1.77 (95% CI 1.08–11.25), started to decrease in 2012 (the 8th year after the ban on AA-CHPs), and reached 1.00 in 2014–2015 based on the speed of 19% (95% CI −2–36%) per year (Figure 3A). In contrast, middle-aged men with a CDAE of more than 150 mg, the aHR increased from 1.51 (95% CI 0.36–6.39) in 2005–2007 to 2.57 (95% CI 0.90–7.31) in 2008–2010 and then declined to 1.30 (95% CI 0.48–2.49) in 2014–2016. The slope-change model showed that the aHR kept at 2.10 (95% CI 0.97–3.24) and decreased in 2009 (the 5th year after the ban on AA-CHPs; Figure 3B). The aHR decreased by 6% (95% CI −7–14%) per year and will reach 1.00 in 2020–2021.

In middle-aged women (40–59 years) with a CDAE of 1–150 mg, the aHRs substantially decreased from 3.30 (95% CI 1.31–8.34) in 2005–2007 to 0.77 (95% CI 0.36–1.68) in 2014–2016. The slope-change model showed that the aHR kept at 2.15 (95% CI 1.32–3.50), started to decrease in 2013 (the 9th year after the ban on AA-CHPs), and reached 1.00 in 2015–2016 based on the speed of 28% (95% CI −2–48%) per year (Figure 3C). Among middle-aged women having CDAE of more than 150 mg, the aHRs significantly decreased from 4.66 (95% CI 1.61–13.46) in 2005–2007 to 2.09 (95% CI 0.92–4.73) in 2014–2016. The slope-change model revealed that the aHR kept at 3.92 (95% CI 1.55–9.87), started to decrease in 2011 (the 7th year after the ban on AA-CHPs), and reached 1.00 in 2016 based on the speed of 22% (95% CI 1–38%) per year (Figure 3D).

### 3.5. Latency period in the older population

In the aged population (60 and 79 years), the aHRs among different time periods were not obvious in the subgroups of participants stratified by either sex or CDAE (Table 3, Figure 3). The selected proportional hazard models showed that the aHRs between 2005 and 2016 were 1.52 (95% CI 1.18–1.95; Figure 3E), 2.29 (95% CI 1.46–3.59; Figure 3F), 1.45 (95% CI 1.18–1.77; Figure 3G), and 2.26 (95% CI 1.57–3.26; Figure 3H).

## 4. Discussion

By investigating a nationwide cohort, our study revealed that the risk of UTUC decreased in the 7th year after the ban on AA in Taiwan. The latency period between the latest AA exposure and the identification of UTUC varied with sex, dose of exposure, and age. The risk of UTUC decreased in the 8th year after the ban of AA in middle-aged men who had exposure doses of 1–150 mg. Likewise, the risk of UTUC started to decrease in the 9th and 7th years after the ban on AA in middle-aged women who had exposure doses of 1–150 mg and >150 mg, respectively. In addition, the risk of UTUC disappeared approximately 10 years after the ban on AA in these women. No significant change in risk was observed in people aged ≥60 years.

Several studies have investigated the carcinogenesis of UTUC by AA. When the cells metabolize AA, the main components of AA, AA I and II become a reactive intermediate which binds to DNA. Then, aristolactam-DNA adducts can be detected in the DNA of cells exposed to AA (24). Besides, aristolactam-DNA adducts have been also identified in renal tissues from the patients with Balkan endemic nephropathy (4), and Taiwanese UTUC patients (6). In a human *p53* knock-in mice embryonic fibroblast cell line (Hupki), the signature mutation pattern, A:T to T:A transversion, was observed after exposure to AA (25). This mutation pattern have been also discovered in the animal models (26, 27), and urothelial carcinoma patients who ever used AA (28). In addition, a high mutation burden was observed in AA-related UTUCs when compared with smoking-related UTUC (29). Although it was difficult to distinguish mutated driver or passenger genes among the average 524 genes in each AA-related UTUC, several driver genes were frequently identified, including *TP53* (58%), *NRAS* (15%), *FGFR3* (8%), and *HRAS* (4%) (29).

Although the health authorities banned on AA according to the molecular (4, 6, 30) and epidemiological evidence (7), the incidence of UTUC seemed not to decrease immediately in the areas with nationwide exposure to AA (31). This implied that AA might have a longer latency period for UTUC. Recently, Jhuang et al. reported that the number of UTUC cases started to decrease after 2010 in Taiwan (12). Accordingly, our Taiwanese cohort was the ideal model to investigate the latency period of AA-induced UTUC based on the below reasons, (1) nationwide exposure of AA, (2) the sharp interruption date of AA usage, (3) the nationwide health insurance which reimbursed AA-CHPs, and (4) the identification of the trend changes of UTUC incidence.

Previous population-based studies revealed that exposure to higher doses of AA resulted in a higher risk of UTUC (7, 20). Our analysis revealed the same finding and further identified the decreasing trend of UTUC risk with time after the ban of AA usage was obvious in the population who took CDAE of >150 mg of AA. This finding indicated that AA could be considered the major carcinogen in people exposed to a high dose of AA. When the responsible carcinogen, AA, was removed, the causal relationship was clearly confirmed by the decreasing risk of UTUC in Taiwan. Although there is no available literature reporting the risk change of UTUC in patients with different dosages of AA exposure, the evidence from studies considering the behavior of cigarette smoking cessation might support a similar phenomenon. The Korean National Health Insurance (NHI) Service database reported that a more reduced risk of all cancers was noted in the smoking reducers of heavy to light smokers than in moderate to light smokers (32).

Our results did not reveal a significant decreasing trend in UTUC risk in elderly people. The possible reasons include: (1) the competing carcinogens for the development of UTUC and (2) the difference in latency periods between long-term and short-term exposure to AA. Since several carcinogens, such as AA (3, 6, 7), smoking (33), and arsenic (20), may contribute to UTUC occurrence, the aged people were logically exposed to more kinds or higher cumulative dosages of carcinogens than the young or middle-aged people. No literature has disclosed the effects of age on the trend of risk of AA-related disease until now, but other carcinogens, such as cigarette smoking, have addressed this issue (34). Using the US National Health Interview Survey for 1997 to 2014, which was linked to the National Death Index, Thomson et al. observed that smokers who quit smoking at an older age had a lower cancer mortality risk than those who quit smoking at a younger age (34).

Unlike in the United States (35), the UTUC risk was not significantly higher in men than in women in Taiwan (6, 31). Our studies revealed that Taiwanese women had taken more AA-CHPs than men. AA-induced UTUCs were more frequently observed in women in a previous cohort (36). The current investigation revealed a clear decreasing trend in UTUC risk after the ban of AA in middle-aged women regardless of AA dose. Although decreasing trends were also observed in middle-aged men regardless of AA dose, men may have multiple carcinogens. For example, cigarette smoking, a common risk factor for UC, was significantly higher in men (approximately 25–40%) than in women (4%) in Taiwan (37). Chen et al. also reported that male patients with UTUC smoked more than female patients in a Taiwanese cohort (36). Once AA was banned, a significant proportion of men were still exposed to carcinogens from cigarette smoking, which might mitigate the decreasing trend of UTUC.

This study has several limitations. First, only the herbal prescriptions reimbursed by the Taiwan NHI between 2000 and 2005 were used for the dosage calculation of AA exposure. We would underestimate the doses because the AA-CHPs prescribed before 2000 or not reimbursed by NHI were not included in this study. Second, some participants might be exposed to AA from the unidentified sources other than the AA-CHP reimbursed by NHI after 2005. Since our finding revealed the ban of AA reduced the incidence of UTUC, these unidentified sources of AA would be minor. Third, even Taiwan NHI covers more than 98% of the population and offers convenient medical access, it may not avoid underdiagnosed given their asymptomatic nature. Patients with either gross or microscopic hematuria can easily access medical resources. This mitigated the selection bias among populations with different doses of AA. Fourth, few UTUC cases in some subgroups might make the estimation of hazard ratio or hazard change imperfect. Even when we used the national database, the rare disease (cancer) still limited the total number of available cases for the analysis. Fifth, not all known carcinogens have been identified. Exposure to AA and arsenic, but not smoking behavior, was measured in this study. This was a natural limitation of the national database, which included only disease codes and prescriptions. The lack of information on smoking behavior made the analysis of this carcinogen impossible. In addition, some possible confounding factors, such as family history, occupation, and education level were not included in this study.

## 5. Conclusion

After the ban on AA was implemented, a decreasing trend in the incidence of UTUC was observed, especially in middle-aged women with AA exposure and in men with moderate doses of AA exposure. The latency period between AA exposure and UTUC development varied with age, dose of AA exposure, and sex.

## Data availability statement

The data analyzed in this study is subject to the following licenses/restrictions: The data that support the findings of this study are available from the Health and Welfare Data Science Center but restrictions apply to the availability of these data, which were used under license for the current study, and so are not publicly available. Data are however available from the authors upon reasonable request and with permission of the Ministry of Health and Welfare, Executive Yuan, Taiwan. Requests to access these datasets should be directed to https://www.apre.mohw.gov.tw/.

## Ethics statement

The studies involving human participants were reviewed and approved by National Taiwan University Hospital. Written informed consent for participation was not required for this study in accordance with the national legislation and the institutional requirements.

## Author contributions

Conceptualization and writing: J-RJ and C-HC. Methodology and data interpretation: J-RJ, W-CL, and C-HC. Investigation: J-RJ, P-CC, and T-CH. Statistical analysis: J-RJ and W-CL. Writing review and editing: C-HC, Y-SP, and W-CL. All authors contributed to the article and approved the submitted version.
