# Supplementary material for: Latency period of aristolochic acid-induced upper urinary tract urothelial carcinoma
Source: Front Public Health. 2023 Mar 9;11:1072864. doi: 10.3389/fpubh.2023.1072864 (PMC10034135; doi:10.3389/fpubh.2023.1072864)
Supplement: Supplementary file 1 [file Data_Sheet_1.pdf]

Figure S1. Arsenic-endemic areas (left part: red) and urbanized areas (right part: red) in Taiwan.

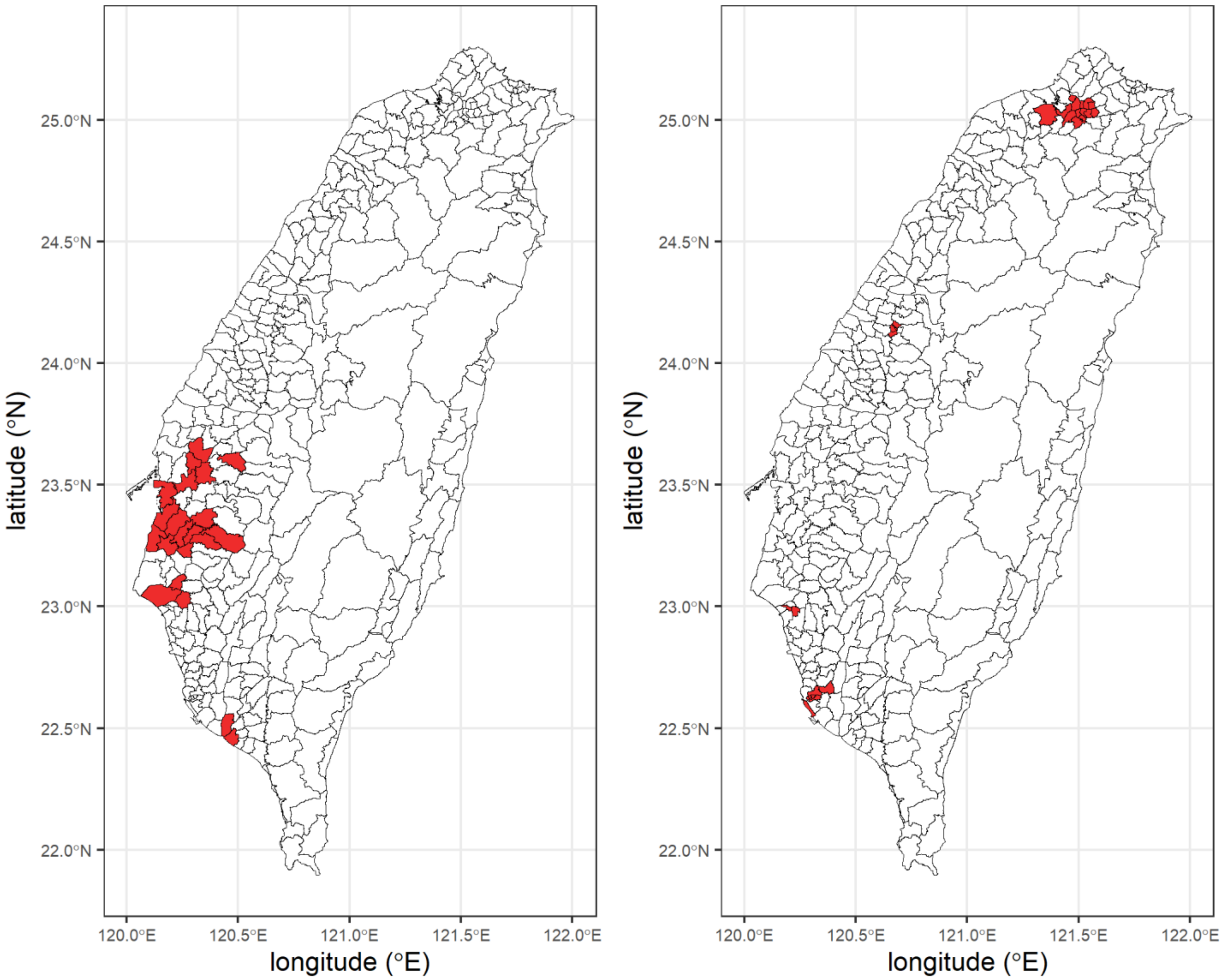

Table S1. The source, route, dosage and duration of aristolochic acid exposure among regions

Regions		Belgium	Balkan	Taiwan
Aristolochic acid	Cohort at risk	Young women	Population at selected villages	All population
	Source	Slimming herbal product	Flour	Traditional herbal product
	Route	Oral	Oral	Oral
	Dosage	Maximal daily dose: 0.025 mg/kg bodyweight	Not reported	Cumulative doses between 2000 and 2005: 0-1 mg (68.29%), 1-150 mg (27.59%), >150 mg (4.12%)
	Duration	13 months (average)	Decades	Intermittent exposure before 2005
	Reference	1, 2		This study

References: 1. Nortier JL, et al. N Engl J Med 2000; 342: 1686-92; 2. Debelle FD, et al.J Am Soc Nephrol 2002; 13: 431-6
